# Search of antimicrobial lactic acid bacteria from *Salmonella*-negative dogs

**DOI:** 10.1186/s12917-021-03070-x

**Published:** 2022-01-03

**Authors:** Estrella Jimenez-Trigos, Marion Toquet, Marta Barba, Ángel Gómez-Martín, Juan J. Quereda, Esther Bataller

**Affiliations:** 1 Microbiological Agents Associated with Animal Reproduction (ProVaginBIO) Research Group, Departamento Producción y Sanidad Animal, Salud Pública Veterinaria y Ciencia y Tecnología de los Alimentos, Facultad de Veterinaria, Universidad Cardenal Herrera-CEU, CEU Universities, Carrer Tirant lo Blanc, 7, 46115 Alfara del Patriarca, Valencia, Spain; 2 Research Group Intracellular Pathogens: Biology and Infection, Departamento Producción y Sanidad Animal, Salud Pública Veterinaria y Ciencia y Tecnología de los Alimentos, Facultad de Veterinaria, Universidad Cardenal Herrera-CEU, CEU Universities, Valencia, Spain

**Keywords:** Lactic acid bacteria, *Salmonella*, Probiotics, *Ligilactobacillus salivarius*, *Lactobacillus*, Dogs

## Abstract

**Background:**

Salmonellosis is one of the most important food-borne zoonotic disease affecting both animals and humans. The objective of the present study was to identify gastrointestinal (GI) lactic acid bacteria (LAB) of canine-origin from *Salmonella*-negative dogs’ faeces able to inhibit monophasic *Salmonella *Typhimurium previously isolated from dogs’ faeces, in order to be used as a potential probiotic in pet nutrition.

**Results:**

Accordingly, 37 LAB were isolated from *Salmonella*-negative dogs’ faeces and tested against monophasic *S. *Typhimurium using the spot on lawn method out of which 7 strains showed an inhibition halo higher than 2.5 cm. These 7 strains were also tested with the co-culture method and one showed the greatest inhibition value *(p < 0.05*). Subsequently, the isolate was identified through 16S rRNA sequencing and sequence homology and designated as *Ligilactobacillus salivarius (L. salivarius)*. LAB from *Salmonella*-positive dogs were also identified and none was the selected strain. Finally, to identify the mechanism of inhibition of *L. salivarius*, the supernatant was analyzed, and a dose response effect was observed.

**Conclusions:**

It is concluded that the canine-origin *L. salivarius*, could possess some in vitro functional attributes of a candidate probiotic and could prevent monophasic *S. *Typhimurium colonization or inhibit its activity if the infection occurs.

**Supplementary Information:**

The online version contains supplementary material available at 10.1186/s12917-021-03070-x.

## Background

Antibiotics have been widely used in small animals’ veterinary practices for treatment of some medical conditions. However, overuse of antimicrobials in pets happens and during the last decades we have witnessed how the indiscriminate use of antibiotics has resulted in the emergence of multidrug-resistant strains even in pets [1–5]. It is estimated that over 70% of the bacteria responsible for healthcare associated infections are resistant to at least one of the antibiotics used worldwide as a first-line therapy [6, 7]. Antimicrobial resistance (AMR) is a global threat worldwide. According to the tenth ESVAC report (European Monitoring of Veterinary Antimicrobial Consumption), the consumption of antimicrobials in Spain is one of the highest among European countries [8].

Antibiotic resistance in enteric pathogens such as *Salmonella* spp. is a major concern for public health safety. According to The European Food Safety Authority [9], salmonellosis is the second most commonly reported gastrointestinal infection in humans and in 2019, 87,923 confirmed cases were reported, many of which are due to antibiotic-resistant *Salmonella* spp.

Several studies have been conducted worldwide to assess the prevalence of *Salmonella* spp. in clinically healthy and diarrheic dogs [10–14]. However, prevalence in clinically healthy dogs varies notably and may even be different depending on the country [15]. It has been shown that healthy dogs can harbour *Salmonella* spp. and eating contaminated foods, including unprocessed or raw dog food, especially raw meat, has been related as one of the most important risk factors of *Salmonella* spp. carriage [10, 13]. Several authors have reported antibiotic resistance in *Salmonella* isolates from dogs [10, 11, 15, 16] and, as they are often in close contact with humans, they can be a source or a recipient of resistant bacteria such as *Salmonella* spp. [17]. Faced with this complex situation, the Spanish National Plan Against Antibiotic Resistance (PRAN) was approved in 2014 with the objective of reducing the risk of dissemination of resistance to antibiotics and has provided numerous achievements, including the decrease of 7.2% of antibiotics consumption in human health, and 14% in veterinary medicine. However, the lack of alternative treatments could be one of the biggest problems of public health.

A recent study made in Spain showed a relationship between LAB content and the absence of *Salmonella* spp. in dogs’ faeces [14]. LAB are the microorganisms most commonly used as probiotics which can be used to prevent infections or as alternatives of antibiotics. Certain probiotic strains of *Lactobacillus* have been reported to be effective against microbial Gram-negative pathogens involved in diarrhoea, gastroenteritis, urovaginal infections and inflammatory bowel disease [18, 19] and also can exert a beneficial effect on the intestinal microbiota increasing the number of lactobacilli and modulating the physiology and immunity parameters of dogs microbiota [20]. Several studies have reported a reduction of *Salmonella* spp. in both chicken and poults after using probiotics [21, 22]. However, studies using probiotics of canine origin, from different dogs breeds, are very limited and this has become of great interest in the scientific canine community [23]. To the best of our knowledge, no studies have demonstrated if GI LAB from *Salmonella*-negative dogs are able to inhibit this pathogen and consequently protect them against the infection.

In that sense, since a key tool to cope with microbial resistance is prevention, the main objective of our study was to identify antimicrobial LAB from *Salmonella*-negative dogs able to inhibit monophasic *S. * Typhimurium isolated from *Salmonella*-positive dogs. Secondly, this study aimed to find out if the LAB identified in *Salmonella*-negative dogs were isolated from *Salmonella*-positive dogs. Finally, the purpose of this study was to explain if GI LAB with antimicrobial activity from *Salmonella*-negative dogs could justify the absence of the pathogen or could protect dogs against monophasic *S. *Typhimurium carriage.

## Results

### Obtention of LAB isolates

Thirty-seven GI LAB were randomly selected from different *Salmonella*-negative dogs isolated and grown in Man, Rogosa and Sharpe (MRS) agar plates as it was described in a previous study [14]. LAB were obtained from males and females of different breeds and ages, housed in different environments.

### Inhibition assays in solid and liquid media: spot-on- lawn and co-culture methods

#### Inhibition assays in solid media: spot-on-law method

**Fig. 1:**
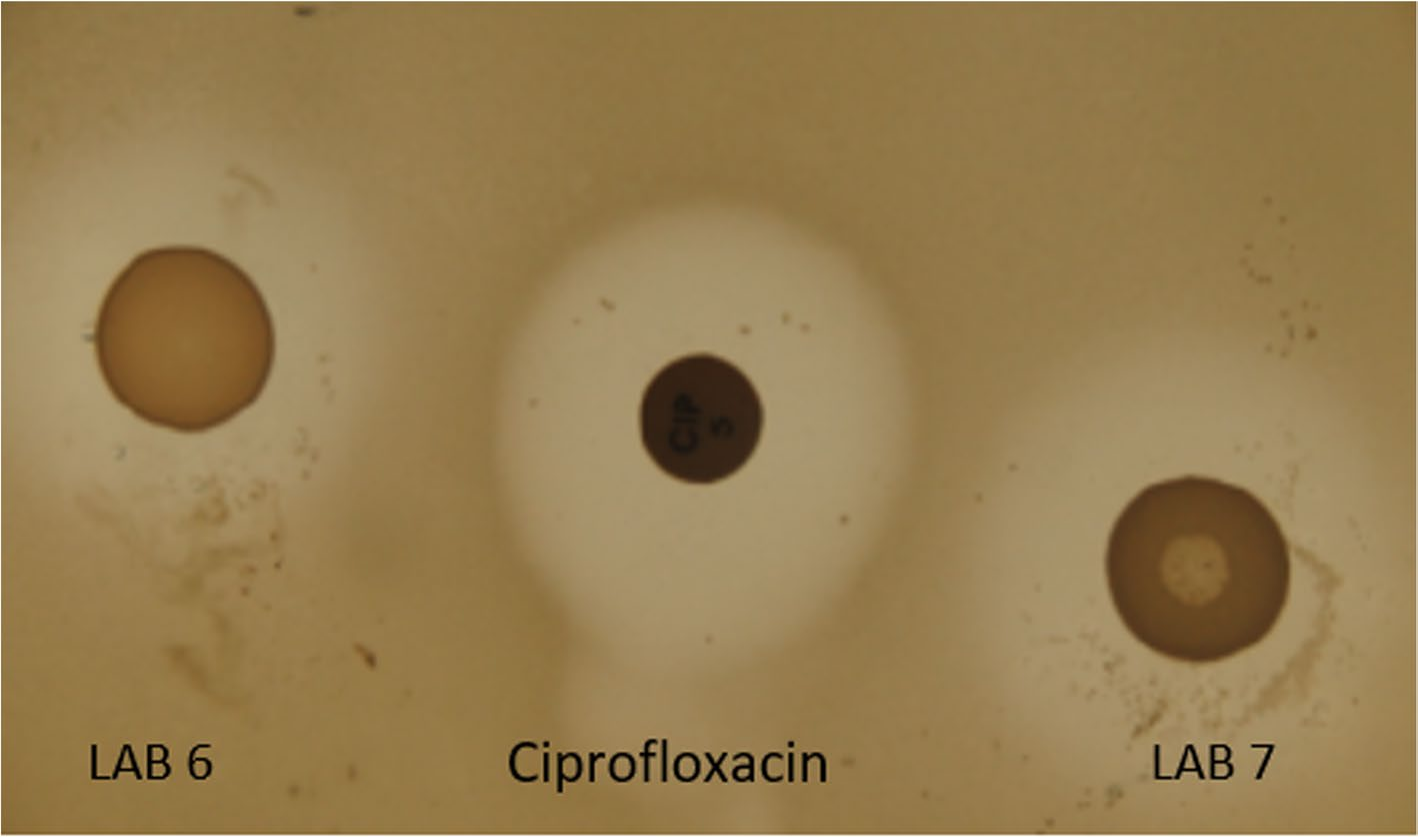
Inhibition halos of LAB 6, 7 and the antibiotic ciprofloxacin with the spot-on-lawn method

Results obtained from the spot-on-lawn technique indicated that the median value of the diameter of the inhibition halos was significantly higher than 1.5 cm (*p < 0.01*). Several LAB were able to inhibit monophasic *S. *Typhimurium with a diameter of inhibition higher than 2.0 cm (Table 1). The greatest inhibition values corresponded to isolates 8, 12, 15, 18, 19, 30, 36. The halo diameter of seven LAB chosen for the next co-culture assay were significantly higher than 2.5 cm (*p < 0.05*). The antibiotic (ciprofloxacin) used as control showed an average inhibition halo of 2.04 cm (Fig. 1).

**Table 1:** Average and standar desviation inhibition halo (cm) of the thirty-seven LAB in the spot-on lawn assay

LAB	Average inhibition halo (cm)	LAB	Average inhibition halo (cm)	LAB	Average inhibition halo (cm)
1	1.800 ± 0.265	13	2.200 ± 0.100	25	1.667 ± 0.577
2	0.900 ± 0.100	14	2.033 ± 0.058	26	1.600 ± 0.600
3	1.600 ± 0.265	15	**3.000 ± 0.100**	27	2.000 ± 0.436
4	1.667 ± 0.289	16	1.933 ± 0.208	28	1.967 ± 0.153
5	2.367 ± 0.252	17	2.200 ± 0.500	29	2.000 ± 0.100
6	1.800 ± 0.346	18	**2.533 ± 0.451**	30	**3.000 ± 0.100**
7	1.800 ± 0.361	19	**2.900 ± 0.200**	31	2.400 ± 0.458
8	**2.850 ± 0.568**	20	2.433 ± 0.058	32	1.933 ± 0.503
9	2.200 ± 0.200	21	2.067 ± 0.306	33	2.100 ± 0.100
10	1.967 ± 1.060	22	2.100 ± 0.265	34	2.367 ± 0.551
11	0.000 ± 0.000	23	2.267 ± 0.551	35	0.000 ± 0.000
12	**2.600 ± 0.100**	24	1.700 ± 0.265	36	**3.100 ± 0.173**
				37	1.533 ± 0.321

#### Inhibition assays in liquid media: co-culture method

**Fig. 2:**
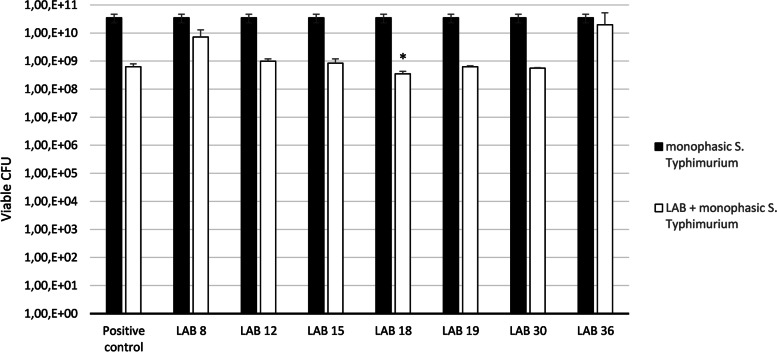
Growth inhibition of monophasic *S.* Typhimurium in XLD by the seven selected LAB strains compared with the control *L. reuteri *Protectis®. The significantly higher logarithmic reduction in monophasic *S. *Typhimurium's growth caused by LAB 18 (MZ602128) is marked by an asterisk * (*p < 0.05*)

In the co-culture method, two parameters were evaluated with these seven selected BAL from the spot-on-lawn method. Firstly, we studied in Xylose Lysine Deoxycholate (XLD) agar the inhibition of monophasic *S. *Typhimurium by LAB compared with the control *L. reuteri *Protectis® (Fig. 2).

**Fig. 3:**
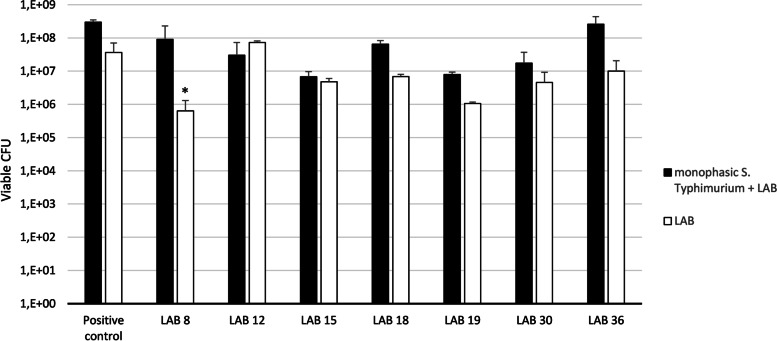
Growth increasement of LAB after incubation with monophasic *S. *Typhimurium in MRS medium compared with the control *L. reuteri *Protectis®. The significantly higher increase in LAB’s growth seen in LAB 8 (MZ602127) is marked by an asterisk (*p < 0.05*)

On the other hand, we studied the survival of these LAB after incubation with monophasic *S. *Typhimurium using MRS plate agar (Fig. 3).

Results of the inhibition assay in XLD medium and in MRS medium indicated that LAB 18, 19, 30 produced a reduction in LOG of monophasic *S. *Typhimurium higher than the positive control *L. reuteri * Protectis® and LAB 8, 18, 36 had an increase growth in MRS also higher than the positive control (Table 2). Wilcoxon test showed that strain 18 had the greatest value of inhibition, which was both higher than the positive control and significantly higher than the other six selected LAB from spot-on-lawn (*p < 0.05*) and strain 8 the greatest value of proliferation (*p < 0.05*).

**Table 2:** Values of inhibition of monophasic *S. * Typhimurium by LAB in XLD medium, and increased growth of LAB after incubation with monophasic *S. *Typhimurium in MRS medium

LAB	reduction in LOG monophasic *S. * Typhimurium (XLD)	increase in LOG LAB (MRS)
**Positive control** ***L. reuteri*** **Protectis®**	**1.74**	**0.91**
**8**	0.68	2.16*
12	1.55	-0.39
15	1.62	0.15
**18**	2.00*	**0.98**
**19**	**1.75**	0.87
**30**	**1.79**	0.58
**36**	0.25	**1.41**

(*) Significantly higher values in LAB 8 and 18 (*p* < 0.05 in both cases)

**Fig. 4:**
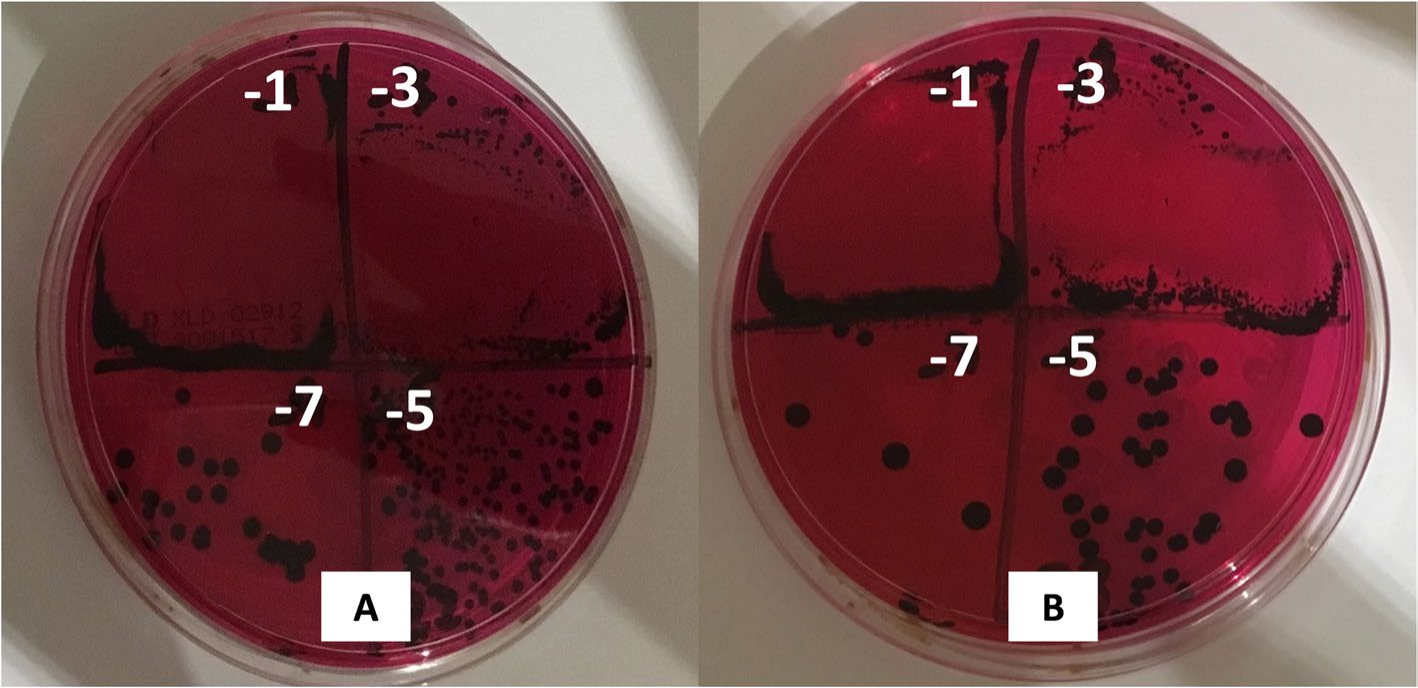
Monophasic *S. *Typhimurium growth in XLD. **A** monophasic *S. *Typhimurium control **B** monophasic *S. *Typhimurium co-incubated with LAB 18 (MZ602128)

**Fig. 5:**
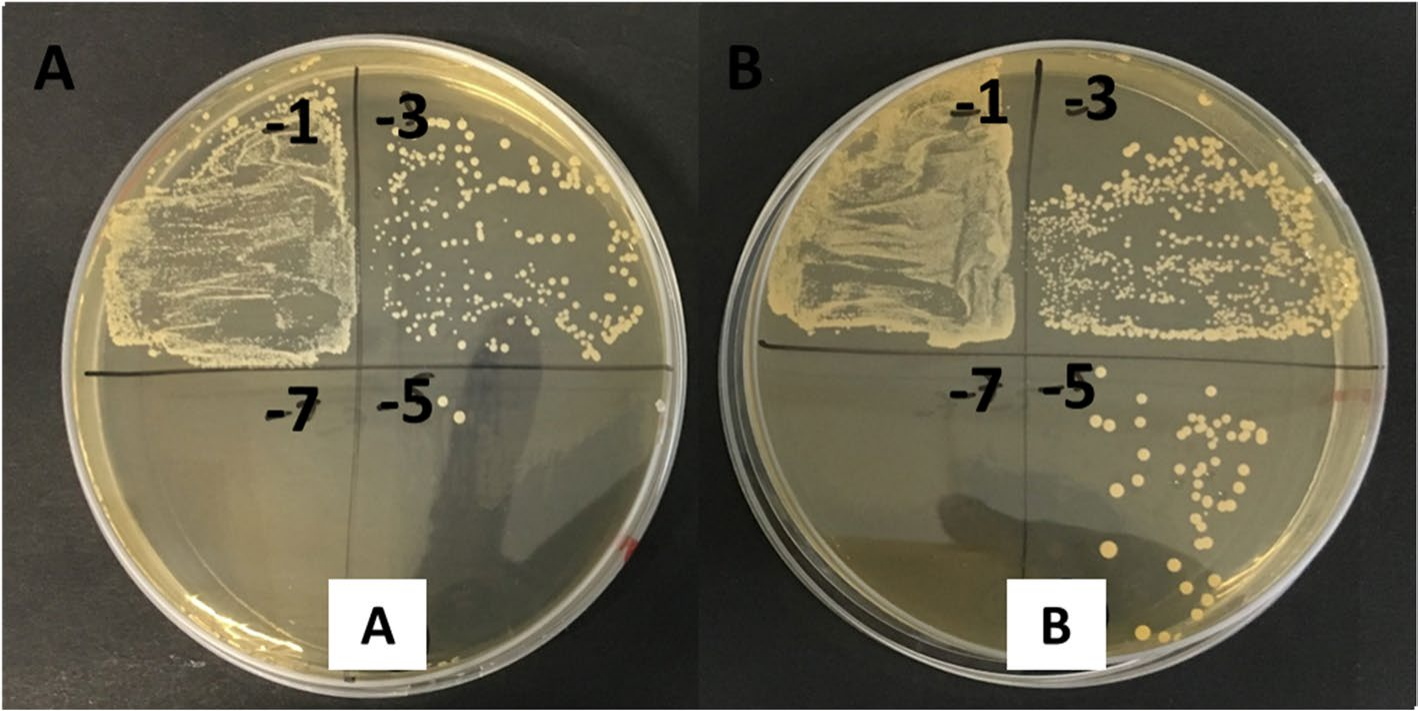
LAB 18 (MZ602128) growth in MRS. **A** monoculture of LAB 18 (MZ602128) **B** LAB 18 (MZ602128) after the co-incubation with monophasic *S*. Typhimurium

Based on these results, LAB 18 was selected as a possible probiotic candidate due to its capacity to inhibit significantly monophasic *S. *Typhimurium in XLD (Fig. 4) and to grow even more in presence of monophasic *S. *Typhimurium in MRS (Fig. 5).

### Antibiotic susceptibility profile of selected LAB and monophasic *S. *Typhimurium strain

Antibiotic susceptibility profiles showed that most of LAB were sensitive to all antibiotics tested. Values demonstrated that the strains were sensitive to beta-lactam antibiotics such as ampicillin, to aminoglycosides such as gentamicin and to other broad-spectrum antibiotics such as chloramphenicol. Only strains 12, 15, 30 and 36 were resistant to some of them and the monophasic *S. *Typhimurium strain used was resistant to ampicillin (Table 3).

**Table 3:** Antimicrobial resistance of selected LAB from spot-on-lawn

LAB/AB	Antimicrobial susceptibility										
	AMP10	CTX30	CAZ30	GM10	ND30	CIP5	AZM15	TGC15	SXT25	CT10	C5
8	S	S	S	S	S	S	S	S	S	S	S
12	S	S	S	S	S	R	S	S	S	S	S
15	R	S	S	S	S	R	S	S	S	S	S
18	S	S	S	S	S	S	S	S	S	S	S
19	S	S	S	S	S	S	S	S	S	S	S
30	S	S	S	S	S	R	S	S	S	S	S
36	S	S	S	S	S	R	S	S	S	S	S
Monophasic *S. *Typhimurium	R	S	S	S	S	S	S	S	S	S	S

*AMP *Ampicillin, *CTX* Cefotaxime, *CAZ* Ceftazidime, *GM* Gentamicin, *ND* Nalidixic acid, *CIP *Ciprofloxacin, *AZM *Azithromycin, *TGC *Tigecycline, *SXT *Trimethoprim sulfamethoxazole, *CT *Colistin, *C* Chloramphenicol

### Survival after exposition to different conditions

All the LAB selected from the co-culture method grew in MRS broth at 25, 30 and 40 °C, under anaerobiosis. They were also able to grow after conventional freeze-drying and after storage at − 20 and − 80 °C for 30 days. Under such conditions, the bacterial viability after incubation was 89.9% of the one found in the control cultures (MRS broth, 37 °C, anaerobiosis).

### Molecular characterization

Strains that showed better results than the commercial positive control *L. reuteri * Protectis® in the co-culture assay (LAB 8, LAB 18, LAB 19, LAB 30 and LAB 36) (Table 2) were identified by molecular characterization, corrected and aligned by ClustalW with MEGA7 (Supplemental file). Based on the sequence obtained, LAB 8, LAB 18, LAB 19, LAB 30 and LAB 36, all obtained from *Salmonella*-negative dogs, were identified as *Ligilactobacillus animalis (*MZ602127), *Ligilactobacillus salivarius (*MZ602128) *(*basionym: *Lactobacillus salivarius), Ligilactobacillus salivarius (*MZ602129)*, Lactobacillus reuteri (*MZ602130) and *Ligilactobacillus salivarius* (MZ602131)*,* respectively (Table 4).

**Table 4:** GenBank accession number(s) for nucleotide sequence

Bacteria Internal code	Submission number	Accesion number to GenBank
**LAB 8**	SUB10053349	MZ602127
**LAB 18**	SUB10053349	MZ602128
**LAB 19**	SUB10053349	MZ602129
**LAB 30**	SUB10053349	MZ602130
**LAB 36**	SUB10053349	MZ602131
**Dog 1**	SUB10053349	MZ602132
**Dog 2**	SUB10053349	MZ602133
**Dog 3**	SUB10053349	MZ602134
**Dog 4**	SUB10053349	MZ602135
**Dog 5**	SUB10053349	MZ602136
**Dog 6**	SUB10053349	MZ602137
**Dog 7**	SUB10053349	MZ602138
**Dog 8**	SUB10053349	MZ602139
**Dog 9**	SUB10053349	MZ602140
**Dog 10**	SUB10053349	MZ602141
**Dog 11**	SUB10053349	MZ602142
**Dog 12**	SUB10053349	MZ602143

In addition, LAB isolated from *Salmonella*-positive dogs were also sequenced. LAB identified from *Salmonella*-positive dogs were *Enterococcus faecium (3), Enterococcus faecalis* (3), *Enterococcus gilvus* (2), *Limosilactobacillus ingluviei* (2) and *Lactobacillus johnsonii (1), Lactobacillus crispatus (1)* (Table 4)*.*

### Antimicrobial activities of the cell free supernatant

**Fig. 6:**
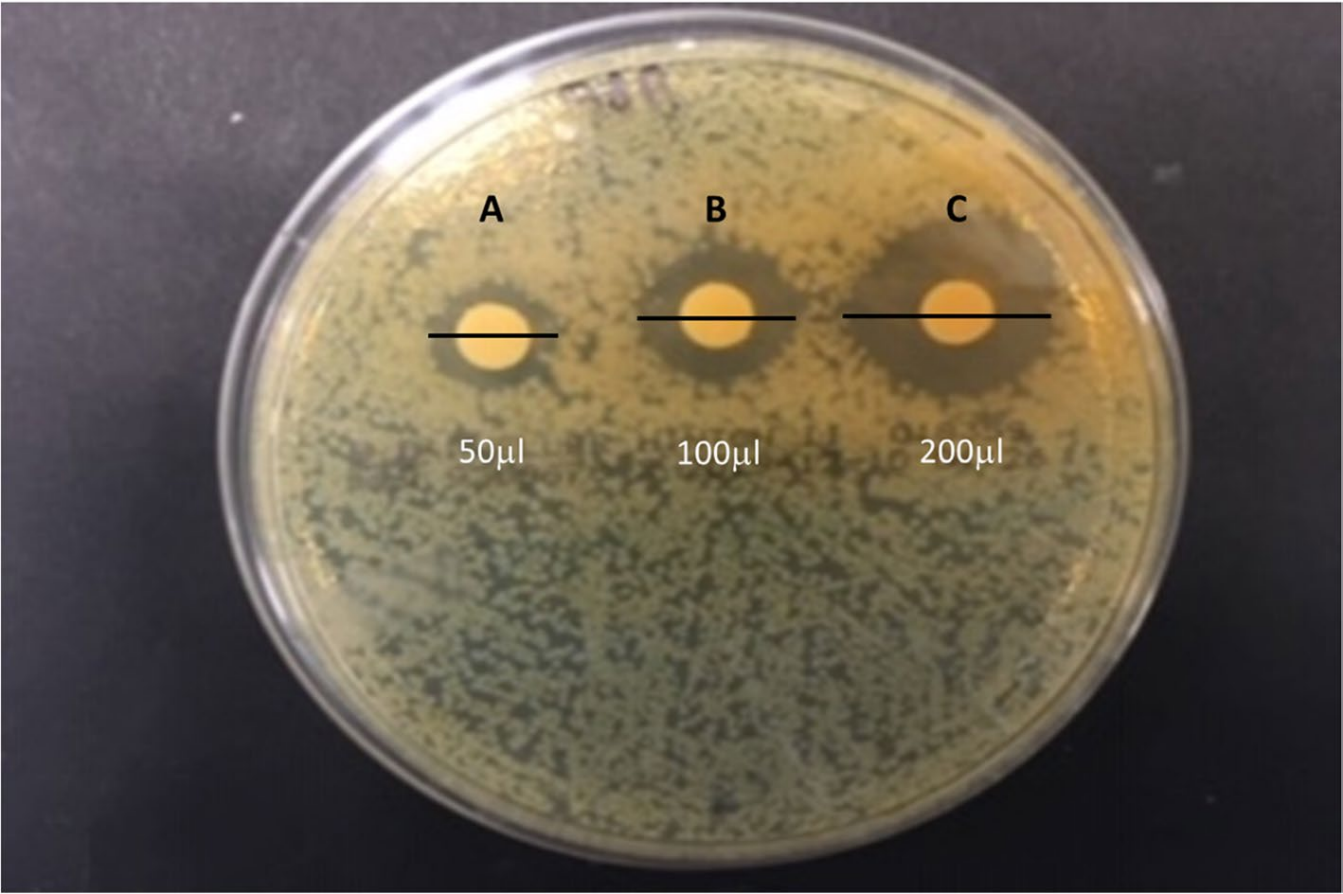
Agar plate showing the inhibitory activity of filtered supernatant of *L. salivarius* (LAB 18) isolated from *Salmonella*-negative dogs against monophasic *S. *Typhimurium: **A** 50 μL of *L. salivarius* cell free supernatant, **B** 100 μL of *L. salivarius* cell free supernatant and **C** 200 μL of *L. salivarius* cell free supernatant

LAB 18 (MZ602128), identified as *Ligilactobacillus salivarius* was used for the study of cell free supernatant. Results showed that different concentrations of supernatant containing an active compound, were able to inhibit monophasic *S. *Typhimurium in Mueller Hinton Plate Agar. A dose-dependent response was clearly observed (Fig. 6).

## Discussion

Nowadays, *Salmonella* is considered one of the four key global causes of diarrheal diseases and it is currently one of the most widely studied bacterial pathogen affecting both domestic and wild animals and humans. Several potential GI pathogens are recognized in dogs, including *Salmonella* spp. [24]. A previous study [14] has suggested that intestinal LAB macroscopic differences observed in *Salmonella*-negative dogs and *Salmonella*-positive dogs could be protecting dogs against *Salmonella* spp. LAB are the most significant groups of probiotic organisms and probiotic supplementations have been successfully used in the prevention and treatment of acute gastroenteritis [25], and their presence could protect against pathogens infections in humans and animals [26, 27]. Different studies have demonstrated the in vitro and in vivo antimicrobial activities of different LAB in dogs [23] or have studied the effects of selected probiotic strains or probiotic mixtures on the microbiome. However, to our knowledge, no previous works have been carried out evaluating the inhibition activity of GI LAB isolated from *Salmonella*-negative dogs against this pathogen and have not identified these LAB strains in *Salmonella*-positive dogs. Based on these considerations, the present work has focused on the screening and selection of the best LAB from *Salmonella*-negative dogs able to inhibit a monophasic *S. * Typhimurium strain isolated in our previous work [14] from a *Salmonella*-positive dog. Firstly, a screening of thirty-seven GI LAB isolated from *Salmonella*-negative dogs were tested in vitro with the spot-on-lawn method. Seven LAB showed an inhibition diameter against monophasic *S. *Typhimurium higher than 2.5 cm and were tested in a co-culture assay in liquid media using commercial *L. reuteri * Protectis® as positive control. In this co-culture study, three LAB (18, 19 and 30) demonstrated the capacity to inhibit monophasic *S. *Typhimurium even more than the commercial control *L. reuteri *Protectis®. These strains were identified by molecular techniques as *Ligilactobacillus salivarius (L.salivarius)* in all three cases and LAB 18 showed, significantly, the higher logarithmic reduction in monophasic *S. *Typhimurium, which was both higher than control and significantly higher than the other six selected LAB from spot-on-lawn (*p < 0.05*). Secondly, in the co-culture study, we analyzed the capacity of the seven selected LAB from the spot-on-lawn assay, to grow more in MRS incubated with monophasic *S. *Typhimurium than as monocultures. We found that three strains (LAB 8: MZ602127, LAB 18: MZ602128 and LAB 30: MZ602130) had this capacity compared with the commercial positive control *L. reuteri *Protectis®. LAB 8 (MZ602127) was identified as *Ligilactobacillus animalis* and LAB 30 (MZ602130) as *Limosilactobacillus reuteri.* Statistically, *L. animalis* showed the greatest value of proliferation in comparison with the other six LAB. Our results are in accordance with an in vivo study which has demonstrated that, when *L. animalis* was added, a reduction of enterococci and an increase of lactobacilli counts throughout was observed, indicating that the administration of *L. animalis* could positively influence composition and metabolism of the intestinal microbiota [28].

Several studies have demonstrated the probiotic attributes of *L. reuteri* and *L. animalis* in dogs [19, 28–30]. Both strains have been identified in canine milk and their probiotic potential was evaluated through different assays, including survival in conditions that resemble those existing in the GI tract, production of antimicrobial compounds, adherence to intestinal mucin, degradation of mucin and pattern of antibiotic sensitivity [31]. Regarding the digestive tract*, L. reuteri* and *L. animalis* inhabit commonly all parts of the dog intestine together with *L. salivarius* and other *Lactobacillus* species [32]*.*

Considering that LAB 18 (MZ602128) produces, significantly, the higher logarithmic reduction in monophasic *S. *Typhimurium's growth in XLD and had a great value of proliferation in MRS, producing an increase in LOG higher than the positive control, and has been identified in three of the five LAB studied in *Salmonella*-negative dogs, this strain was selected as the candidate as probiotic for dogs.

Several studies have demonstrated that *L. salivarius* is part of the canine intestine health [33] and is one of the probiotics available as over-the-counter supplements for dogs [34]. Together with other *Lactobacillus* species, *L. salivarius* is able to modify the dominant indigenous jejunal LAB microbiota [35]. Regarding the antimicrobial capacity of *L.salivarius*, [33] reported that *L. salivarius,* isolated from Border Collie and German Shorthaired Pointer, showed antimicrobial activity towards *Micrococcus luteus.*

Studies made in other animal species have shown that *L. salivarius* from bottlenose dolphin can inhibit the growth of *S. *Enteriditis strains isolated from both marine animals and humans [36]. In piglets, *L. salivarius* strains have been isolated and its antimicrobial effect has been studied against *Salmonella* [37, 38]. Also, it has been found that *L. salivarius* has an antimicrobial activity against *E. coli* and *Klebsiella* in chicken and prevents *S. *Enteriditis colonization [39, 40]. Also, other studies showed that *L. salivarius* could eliminate *S. *Enteriditis from chickens after an oral challenge of the pathogen on day 1 [40]. *L. salivarius* was also proposed as a probiotic product for administration during the feeding of calves for its capacity of aggregation and its good yield [41]. Moreover, it was seen in cattle that *L. salivarius* exhibits remarkable anti-*salmonella* activities with total inhibition of *Salmonella* spp. after 18 h of co-incubation [42].

To our knowledge, in dogs, no study has demonstrated the effect of *L. salivarius* against *Salmonella.* O’Mahony et al. [43], revealed that some LAB, specifically *Bifidobacterium animalis* AHC7, has significant potential for improving canine gastrointestinal health but no *L. salivarius* was identified as a potential probiotic.

Regarding probiotics attributes, *L. salivarius* has been extensively studied [44] as well as *L. reuteri* and *L. animalis*. These LAB are included in the list of taxonomic units proposed for qualified presumption of safety (QPS) status from EFSA and detailed toxicological study was performed following the FAO/WHO recommendations [45]. In addition to its safety and functionality, a probiotic candidate must survive in GI conditions and remains several years in the GI tract of dogs [46].

Taking into account that all LAB from *Salmonella*-negative dogs were identified as *L. salivarius*, *L. reuteri* or *L. animalis*, it is reasonable to think that these strains identified in *Salmonella*-negative dogs may be protecting animals against *Salmonella* colonization and infection.

In *Salmonella*-positive dogs, most of the LAB identified belonged to the * Enterococcus* genus. Rinkinen et al. [47] demonstrated that two strains of *E. faecium* significantly enhanced the adhesion of *Campylobacter jejuni*, up to 134.6 and 205.5%. This suggest that *E. faecium* may thus favor the adhesion and colonization of *C. jejuni* in the dog’s intestine, making it a potential carrier and possibly a source of infection [47]. Another study has demonstrated that the supplementation of *E. faecium* in 12 healthy dogs, kept in households during 18-days, reduces the counts of *Clostridium* spp. while increasing the counts of *Salmonella* spp. and *Campylobacter* spp. in the majority of dogs [48]. In another work published by Rinkinen et al. [47], the ability of certain LAB to inhibit the adhesion of selected canine and zoonotic pathogens was evaluated and they observed that the adhesion of *S. *Typhimurium was not significantly affected by any of the LAB tested; however, *L. reuteri, L. animalis* and *L. salivarius* reduced the counts of monophasic *S. * Typhimurium as previously reported.

One of the main arguments for the use of probiotics in preventing and combating digestive disorders in animals is the inhibition of potential pathogenic bacteria by producing a variety of inhibitory substances [26, 49]. In that sense, to identify the active substance from LAB 18, able to inhibit monophasic *S. *Typhimurium, a preliminary study was carried out in our work. Results showed a clear monophasic *S.* Typhimurium inhibition when different supernatant concentrations were employed. To the best of our knowledge, four reports mention bacteriocin production in *L. salivarius.* Arihara et al. [49] isolated from Japanese grass leaves bacteriocins from *L. salivarius* and Ocaña et al. [50] worked with human vaginal sample bacteriocins. Robredo et al. [51] detected a bacteriocin activity in 61% of the isolates recovered from fecal samples of 20 pigs, and all bacteriocin-producing *L. salivarius* isolates strongly inhibited the growth of *Staphylococcus aureus* and *Staphylococcus epidermidis,* but none of these bacteriocin-producing *L. salivarius* isolates showed growth inhibition activity against *Enterococcus* spp., *Bacillus* spp., or *E. coli* and they were not tested towards *S.* Typhimurium. As far as we know, few studies have identified the inhibitory substances, produced by selected *L. salivarius* strains, able to inhibit *S. *Typhimurium [52].

## Conclusions

We have identified a potential antimicrobial LAB against monophasic *S. *Typhimurium from *Salmonella*–negative dogs, not isolated in *Salmonella*–positive dogs. This LAB, identified as *Ligilactobacillus salivarius* could prevent monophasic *S. *Typhimurium colonization, protecting dogs against *Salmonella* infection or inhibiting its activity if the infection occurs. Further in vitro and in vivo studies must be carried out to evaluate the probiotic attributes and to identify the active compound responsible of the inhibition.

## Material and methods

### Obtention of LAB isolates

Obtention of LAB from *Salmonella*-positive dogs and *Salmonella*-negative dogs was carried out as previously described [14]. As it was indicated in the previous work, *Salmonella*-positive dogs were animals where *Salmonella* spp. were isolated from their faeces while in *Salmonella*-negative dogs no *Salmonella* spp. were found. All animals were handled according to the principles of animal care published by Spanish Royal Decree 53/2013 [53]. Sampled collection was approved by the Ethics Committee and Animal Experimentation of UCH-CEU University.

Samples were inoculated in the MRS agar and incubated 24–48 h in anaerobic conditions at 37 °C. After incubation, morphologically different colonies obtained from *Salmonella*-positive dogs and *Salmonella*-negative dogs were tested as Gram-positive bacteria using the Gram staining method and as catalase positive and were frozen at − 80 °C and stored until their use.

Moreover, data from each dog were also collected by a questionnaire that included data related with the environment: where animals were housed, breed, age, gender, diet or type of food, contact with other animal species and the source of water. All questionnaires were completed and submitted together with the samples to the laboratory.

### Inhibition assays in solid and liquid media: spot-on-lawn and co-culture methods

Monophasic *S. * Typhimurium strain used for inhibition assays in this study was isolated from dogs’ faeces in the same previous work [14] and spot-on-lawn and co-culture assays were performed in three independent experiments. Control medium was carried out in both assays.

### Inhibition assays in solid media: spot-on-lawn method

Thirty-seven LAB from *Salmonella*-negative dogs were selected for inhibition assays. LAB were tested by the spot-on-lawn method and the technique was adapted from the method described by Harris et al. [54]. For this method, 5 mL of an overnight LAB culture in liquid MRS were spotted onto the surface of an MRS agar plate. The plates were incubated at 37 °C in anaerobic conditions (24 h). After incubation, MRS agar plates were overlaid with 10 mL of Nutrient soft agar (0.80% agar) seeded with an overnight broth culture of monophasic *S. * Typhimurium. The overlay was incubated at 37 °C for 24 h and then, the plates were examined for zones of inhibition in the monophasic *S*. Typhimurium cell lawn. Inhibition was determined by measuring the diameter of clear zones (cm) around the LAB spots. For inhibition halos control, an antibiotic disk selected by its inhibition activity against monophasic *S. * Typhimurium (ciprofloxacin) was used. All assays were performed in triplicate.

### Inhibition assays in liquid media: co-culture method

For this experiment, only LAB with the highest inhibition result in the spot-on-lawn method (those with more than 2.5 cm of inhibition halo) were tested. The method was adapted from the previously described by Adetoye et al. [42]. For the co-culture, 10 ml of Todd Hewitt broth (THB) was inoculated with LAB and monophasic *S. * Typhimurium growth at the same OD_600_ in the stationary phase. Two experimental controls, which consisted of a monoculture of each LAB and a monoculture of monophasic *S. * Typhimurium, were set up. All samples were incubated at 37 °C for 24 h. To determine the viable counts (CFU/mL) of both monocultures and the co-cultures, serial ten-fold dilutions were carried out and 25 μL of each dilution were plated on both XLD and MRS agar to control the growth of monophasic *S. * Typhimurium and LAB, respectively. The commercial strain *Lactobacillus reuteri *Protectis® (DSM17938, Casen Recordatori) (*L. reuteri* Protectis®) used in humans against *Salmonella* infection was used as positive control. All assays were performed in triplicate and the log transformation was applied to the average.

### Antibiotic susceptibility profile of selected LAB

The antibiotic susceptibility profile of the LAB isolates was conducted using the agar disk diffusion method established by the Clinical and Laboratory Standards Institute [55]. Antimicrobial agents and concentrations used in this study were those set forth in Decision 2013/653 (European Union 2013:653): ampicillin (10 μg), cefotaxime (30 μg), ceftazidime (30 μg), gentamicin (10 μg), nalidixic acid (30 μg), ciprofloxacin (5 μg), azithromycin (15 μg), tigecycline (15 μg), trimethoprim-sulfamethoxazole (25 μg), colistin (10 μg) and chloramphenicol (5 μg). Antimicrobial susceptibility was tested according to the European Committee on Antimicrobial Susceptibility Testing (EUCAST) guidelines [56]. The source for zone diameters used for interpretation of the test was https://www.eucast.org/clinical_breakpoints/ . Zone diameters were interpreted and categorized as susceptible, intermediate or resistant according to the EUCAST clinical breakpoint tables.

### Survival after exposition to different conditions

The survival of seven LAB, selected from the spot-on-lawn method, after exposure to different conditions for probiotic applications, was tested in MRS broth. The following growth conditions were assayed under anaerobiosis: 25, 30 and 40 °C, freeze-drying and storage at − 20 °C and − 80 °C for 30 days in the presence of 15% (v/v) glycerol. All assays were performed in triplicate. MRS cultures incubated at 37 °C under anaerobiosis were used as controls.

### Molecular characterization

Selected LAB, isolated from *Salmonella-*negative dogs, from the co-culture assay, were used for molecular characterization. Also, LAB isolated from *Salmonella*-positives dogs, were selected for molecular characterization to compare the sequence between positive and negative dogs. LAB were processed for genomic DNA extraction and identified based on PCR amplification and sequencing of 16S rRNA gene using bacterial universal primers (27F 5′- AGAGTTTGATCCTGGCTCAG and 1492R 5′-GGTT ACCTTGTTACGACTT) [23]. The PCR was performed in 25 μl reaction volumes containing 2X Taq Master Mix, 0.25 mM forward primer, 0.25 mM reverse primer and 0.4 ng of genomic DNA and nuclease-free water to make volume 25 μl. Temperature cycling conditions for PCR were as follows: an initial heating of 95 °C for 3 min, followed by 40 cycles of denaturation at 95 °C for 30 s, annealing at 55 °C for 30 s, extension at 72 °C for 90 s and terminating with a 5 min final incubation of 72 °C. The PCR products were examined with electrophoresis on a 1.5% w/v agarose gel, stained by Safe Lab nucleic acid stain. The PCR products were purified, and sequenced and analysed for sequence homology by BLAST (http://www.ncbi.nlm.nih. gov/). The sequences were corrected and aligned by ClustalW with MEGA7: Molecular Evolutionary Genetics Analysis version 7.0 for bigger datasets [23]. Bacterial identification was carried out by comparing the problem sequence with the GenBank database through the Blast application. Only identification with a Query Cover equal to or greater than 98% and with an E value of 0.0 were considered.

### Antimicrobial activities of the cell free supernatant

For this assay, the higher inhibitory LAB able to significantly inhibit monophasic *S.* Typhimurium in the co-culture method was used. A volume of 20 ml culture growing in MRS broth at 37 °C was obtained and centrifugated at 12,000 g for 10 min at 4 °C. Supernatant was neutralized to pH 6.5 with NaOH and filter-sterilised through 0.22 μm pore size filters (Millipore). The bacteriocinogenic activity of the cell-free supernatant was determined in Mueller-Hinton plate agar disk diffusion. The method was adapted from the previously described by Lee et al. [57]. Monophasic *S. *Typhimurium was inoculated on the surface and 50 μl of LAB supernatant was spotted onto the previously inoculated plate. To identify a dose-response effect, different volumes of the supernatant (50, 100 and 200 μl) were concentrated to 50 μl and were impregnated in a blank disk and placed also in the Mueller- Hinton agar plate and the diameter of inhibition was quantified. In all these expermients, filtered supernatant was obtained the same day as bacterial growth was made.

### Statistical analyses

Compliance with the assumption of normality was checked for the diameter of the inhibition halos of the initial thirty-seven LAB isolates by means of the Shapiro-Wilk test. The results showed that the variable did not distribute normally. Accordingly, the nonparametric Wilcoxon one-sample signed rank test (SPSS/ PASW Statistics for Windows, Version 18.0. SPSS Inc., Chicago, IL) was used to show the significant differences between the median of the diameters of the inhibition halos and each reference value. The nonparametric Wilcoxon one-sample signed rank test (SPSS/ PASW Statistics for Windows, Version 18.0. SPSS Inc., Chicago, IL) was also used for co-culture method. An α level of .05 was considered significant for all analyses.

## Supplementary Information

**Additional file 1.**

**Additional file 2.**

## Data Availability

The datasets analyzed during this study are available from the corresponding author on reasonable request. The datasets generated during the current study are available in the GenBank repository, GenBank accession numbers for nucleotide sequences: SUB10053349 LAB8 MZ602127 SUB10053349 LAB18 MZ602128 SUB10053349 LAB19 MZ602129 SUB10053349 LAB30 MZ602130 SUB10053349 LAB36 MZ602131 SUB10053349 dog1 MZ602132 SUB10053349 dog2 MZ602133 SUB10053349 dog3 MZ602134 SUB10053349 dog4 MZ602135 SUB10053349 dog5 MZ602136 SUB10053349 dog6 MZ602137 SUB10053349 dog7 MZ602138 SUB10053349 dog8 MZ602139 SUB10053349 dog9 MZ602140 SUB10053349 dog10 MZ602141 SUB10053349 dog11 MZ602142 SUB10053349 dog12 MZ602143
