# Supplementary material for: Search of antimicrobial lactic acid bacteria from Salmonella-negative dogs
Source: BMC Vet Res. 2022 Jan 3;18:12. doi: 10.1186/s12917-021-03070-x (PMC8767738; doi:10.1186/s12917-021-03070-x)
Supplement: Supplementary file 1 — Additional file 1. [file 12917_2021_3070_MOESM1_ESM.docx]

SUPPLEMENTARY MATERIAL

**Table 1.** Dogs’ characteristics whose LAB were selected for the study.

| **BAL** | **Environment** | **Breed** | **Age** | **Sex** |
| --- | --- | --- | --- | --- |
| 1 | hunting kennels | Braco | puppy | F |
| 2 | household | Golden | adult | F |
| 3 | hunting kennels | Braco | adult | F |
| 4 | hunting kennels | Braco | adult | F |
| 5 | hunting kennels | Bretón | adult | F |
| 6 | household | Golden | adult | F |
| 7 | hunting kennels | Bretón | adult | F |
| 8 | household | Golden | adult | F |
| 9 | animal shelter | cross-breed | adult | M |
| 10 | household | Podenco | adult | F |
| 11 | animal shelter | cross-breed | adult | M |
| 12 | animal shelter | cross-breed | adult | M |
| 13 | animal shelter | cross-breed | adult | M |
| 14 | animal shelter | cross-breed | adult | M |
| 15 | household | cross-breed | adult | F |
| 16 | animal shelter | cross-breed | adult | M |
| 17 | household | cross-breed | adult | M |
| 18 | animal shelter | cross-breed | adult | M |
| 19 | animal shelter | cross-breed | adult | M |
| 20 | animal shelter | cross-breed | adult | M |
| 21 | animal shelter | cross-breed | adult | M |
| 22 | household | cross-breed | adult | M |
| 23 | animal shelter | cross-breed | adult | M |
| 24 | animal shelter | cross-breed | adult | M |
| 25 | animal shelter | cross-breed | adult | M |
| 26 | animal shelter | cross-breed | adult | M |
| 27 | hunting kennels | cross-breed | adult | M |
| 28 | animal shelter | cross-breed | adult | M |
| 29 | animal shelter | cross-breed | adult | M |
| 30 | animal shelter | cross-breed | adult | M |
| 31 | animal shelter | cross-breed | adult | M |
| 32 | household | cross-breed | adult | M |
| 33 | household | cross-breed | adult | M |
| 34 | hunting kennels | BRETÓN | adult | F |
| 35 | household | cross-breed | adult | M |
| 36 | hunting kennels | Teckel | puppy | M |
| 37 | hunting kennels | cross-breed | adult | M |

**Bacterial identification**

LAB 8: *Lactobacillus animalis*.

AAGTGCGTGAGAGTAACTGTTCACGTTTCGACGGTATCTAACCcAGAAAGCCACGGCTAACTACGTGCCAGCAGCCGCGGTAATACGTAGGTGGCAAGCGTTATCCGGATTTATTGGGCGTAAAGGGAACGCAGGCGGTCTTTTAAGTCTGATGTGAAAGCCTTtCGGCTTAACCGGAGTAGTGCATTGGAAACTGGGAGACTTGAGTGCAGAAGAGGAGAGTGGAACTCCATGTGTAGCGGTGAAATGCGTAGATATATGGAAGAACACCAGTGGCGAAAGCGGCTCTCTGGTCTGTAACTGACGCTGAGGTTCGAAAGCGTGGGTAGCAAACAGGATTAGATACCCTGGTAGTCCACGCCGTAAACGATGAATGCTAAGTGTTGGAGGGTTTCCGCCCTTCAGTGCTGCAGCTAACGCAATAAGCATTCCGCCTGGGGAGTACGACCGCAAGGTTGAAACTCAAAGGAATTGACGGGGGCCCGCACAAGCGGTGGAGCATGTGGTTTAATTCGAAGCAACGCGAAGAACCTTACCAGGTCTTGACATCTTCTGACAATCCTAGAGATAGGACTTTCCCTTCGGGGACAGAaTGACAGGTGGTGCATGGTTGTCGTCAGCTCGTGTCGTGAGATGTTGGGTTAAG

LAB 18: *Ligilactobacillus salivarius*.

TCGGATCGTAAAACTCTGTTGTTAGAGAAGAACACGAGTGAGAGTAACTGTTCATTCGATGACGGTATCTAAaCCAGCAAGTCACGGCTAACTACGTGCCAGCAGCCGCGGTAATACGTAGGTGGCAAGCGTTGTCCGGATTTATTGGGCGTAAAGGGAACGCAGGCGGTCTTTTAAGTCTGATGTGAAAGCCTTCGGCTTAACCGGAGTAGTGCATTGGAAACTGGAAGACTTGAGTGCAGAAGAGGAGAGTGGAACTCCATGTGTAGCGGTGAAATGCGTAGATATATGGAAGAACACCAGTGGCGAAAGCGGCTCTCTGGTCTGTAACTGACGCTGAGGTTCGAAAGCGTGGGTAGCAAACAGGATTAGATACCCTGGTAGTCCACGCCGTAAACGATGAATGCTAGGTGTTGGAGGGTTTCCGCCCTTCAGTGCCGCAGCTAACGCAATAAGCATTCCGCCTGGGGAGTACGACCGCAAGGTTGAAACTCAAAGGAATTGACGGGGGCCCGCACAAGCGGTGGAGCATGTGGTTTAATTCGAAGCAACGCGAAGAACCTTACCAGGTCTTGACATCC

LAB 19: *Ligilactobacillus salivarius*.

TTTATTGGGCGTAAAGGGAACGCAGGCGGTCTTTTAAGTCTGATGTGAAAGCCTTCGGCTTAACCGGAGTAGTGCATTGGAAACTGGAAGACTTGAGTGCAGAAGAGGAGAGTGGAACTCCATGTGTAGCGGTGAAATGCGTAGATATATGGAAGAACACCAGTGGCGAAAGCGGCTCTCTGGTCTGTAACTGACGCTGAGGTTCGAAAGCGTGGGTAGCAAACAGGATTAGATACCCTGGTAGTCCACGCCGTAAACGATGAATGCTAGGTGTTGGAGGGTTTCCGCCCTTCAGTGCCGCAGCTAACGCAATAAGCATTCCGCCTGGGGAGTACGACCGCAAGGTTGAAACTCAAAGGAATTGACGGGGGCCCGCACAAGCGGTGGAGCATGTGGTTTAATTCGAAGCAACGCGAAGAACCTT

LAB 30: *Lactobacillus reuteri.*

GCGTGAGTGAAGAAGGGgTTTCGGCTCGTAAAGCTCTGTTGTTGGAGAAGAACGTGCGTGAGAGTAACTGTTCACGCAGTGACGGTATCCAACCAGAAAGTCACGGCTAACTACGTGCCAGCAGCCGCGGTAATACGTAGGTGGCAAGCGTTATCCGGATTTATTGGGCGTAAAGCGAGCGCAGGCGGTTGCTTAGGTCTGATGTGAAAGCCTTCGGCTTAACCGAAGAAGTGCATCGGAAACCGGGCGACTTGAGTGCAGAAGAGGACAGTGGAACTCCATGTGTAGCGGTGGAATGCGTAGATATATGGAAGAACACCAGTGGCGAAGGCGGCTGTCTGGTCTGCAACTGACGCTGAGGCTCGAAAGCATGGGTAGCGAACAGGATTAGATACCCTGGTAGTCCATGCCGTAAACGATGAGTGCTAGGTGTTTGGAGGGTTTCCGCCCTTCAGTGCCGGAGCTAACGCATTAAGCACTCCGCCTGGGGGAGTACGACCGCAAGGTTGAAACTCAA

LAB 36: *Ligilactobacillus salivarius.*

GGATCGTAAAACTCTGTTGTTAGAGAAGAACACGAGTGAGAGTAAaCTGTTCATTCGATGACGGTATCTAACCAGgCAAGTCACGGCTAACTAaCGTGCCAGCAGCCGCGGTAATACGTAGGTGGCAAGCGTTGTCCGGATTTATTGGGCGTAAAGGGAACGCAGGCGGTCTTTTAAGTCTGATGTGAAAGCCTTCGGCTTAACCGGAGTAGTGCATTGGAAACTGGAAGACTTGAGTGCAGAAGAGGAGAGTGGAACTCCATGTGTAGCGGTGAAATGCGTAGATATATGGAAGAACACCAGTGGCGAAAGCGGCTCTCTGGTCTGTAACTGACGCTGAGGTTCGAAAGCGTGGGTAGCAAACAGGATTAGATACCCTGGTAGTCCACGCCGTAAACGATGAATGCTAGGTGTTGGGAGGGTTTCCGCCCTTCAGTGCCGCAGCTAACGCAATAAGCATTCCGCCTGGGGAGTACGACCGCAAGGTTGAAACTCAAaGGAATTGACGGGGGCCCGCACAAGCGGTGGAGCATG

POSITIVE DOG: *Enterococcus faecium.*

AGAAGGGGATAACACTTGGAAACAGGTGCTAATACCGTATAACAATCAAAACCGCATGGTTTTGATTTGAAAGGCGCTTTCGGGTGTCGCTGATGGATGGACCCGCGGTGCATTAGCTAGTTGGTGAGGTAACGGCTCACCAAGGCCACGATGCATAGCCGACCTGAGAGGGTGATCGGCCACAATGGGACTGAGACACGACCCAAACTCCTACGGGAGGCAGCAGTAGGGAATCTTCGGCAATGGACGAAAGTCTGACCGAGCAACGCCGCGTGAGTGAAGAAGGTTTTCGGATCGTAAAACTCTGTTGTTAGAGAAGAACAAGGATGAGAGTAACTGTTCATCCCTTGACGGTATCTAACCAGAAAGCCACGGCTAACTACGTGCCAGCAGCCGCGGTAATACGTAGGTGGCAAGCGTTGTCCGGATTTATTGGGCGTAAAGCGAGCGCAGGCGGTTTCTTAAGTCTGATGTGAAAGCCCCCcGgcctcCAACCGGGGAGGGTCATTGGAAACTGGGAGACTTGAGTGCAGAAGAGGAGAGTGGAATTCCATGTGTAGCGGTGAAATGCGTAGATATATGGAG

POSITIVE DOG: *Lactobacillus ingluviei.*

GCAAGCGTTATCCGGATTTATTGGGCGTAAAGCGAGCGCAGGgCGGTTTTCTAAGTCTGATGTGAAAGCCTTCGGCTTAACCGAAGAAGTGCATCGGAAACTGGATGACTTGAGTGCAGAAGAGGGCAGTGGAACTCCATGTGTAGCGGTGGAATGCGTAGATATATGGAAGAACACCAGTGGCGAAGGCGGCTGCCTGGTCTGTAACTGACGCTGAGGCTCGAAAGCATGGGTAGCGAACAGGATTAGATACCCTGGTAGTCCATGCCGTAAACGATGAGTGCTAGGTGTTGGGAGGGTTTCCGCCCTTCAGTGCCGGAGCTAACGCATTAAGCACTCCGCCTGGGGAGTACGACCGCAAGGTTGAAACTCAAAGGAATTGACGGGGGCCCGCACAAGCGGTGGAGCATGGTGGTTTAATTCGAAGCTACGCGAAGAACC

POSITIVE DOG: *Enterococcus faecalis*.

GTGAAGAAGGTTTTCGGATCGTAAAACTCTGTTGTTAGAGAAGAACAAGGACGTTAGTAACTGAACGTCCCCTGACGGTATCTAACCAGAAAGCCACGGCTAACTACGTGCCAGCAGCCGCGGTAATACGTAGGTGGCAAGCGTTGTCCGGATTTATTGGGCGTAAAGCGAGCGCAGGCGGTTTCTTAAGTCTGATGTGAAAGCCCCCGGCTCAACCGGGGAGGGTCATTGGAAACTGGGAGACTTGAGTGCAGAAGAGGAGAGTGGAATTCCATGTGTAGCGGTGAAATGCGTAGATATATGGAGGAACACCAGTGGCGAAGGCGGCTCTCTGGTCTGTAACTGACGCTGAGGCTCGAAAGCGTGGGGAGCAAACAGGATTAGATACCCTGGTAGTCCACGCCGTAAACGATGAGTGCTAAGTGTTTGGAGGGTTTCCGCCCTTCAGTGCTGCAGCAAACGCATTAAGCACTCCGCCTGGGGAGTACGACCGCAAGGTTGAAACTCAAAGGAATTGACGGGGGC

POSITIVE DOG: *Enterococcus faecium.*

ACTTCCCATTGGAACACGTGTGTAACCCAGGGCATAAGGGGCATGATGATTTGACGTCCTCCCCACCTTCCTCCGGTTTGTCACCGGCAGTCTTGCTAGAATGGCCAACTGAATGATGGCAACTAACAATAAGGGTTGCGCTCGTTGCGGGACTTAACCCAACATCTCACGACACGAACTGACGACCACCATGCACCACCTGGCACTTTGCCCCCGAAGGGGAAGCTCTATCTCTAGAGTGGTCAAAGGATGTCAAGACCTGGTAAGGGTCTTCCCGTTGCTTCCAATTAAACCACATGCTCCACCGCTTGGGCGGGCCCCCGTCAATTCCTTTGAATTTCAACCTTGCGGTCGTACTCCCCAGGGGGAATGGTTAATGGGTTAGCTGCAGCACTGAAGGGCGGAAACCCTCCAACACTTAACACTCATCGTTTACGGCGTGGACTACCAAGGTATCTAATCCTGTTTGCTCCCCACGCTTTCGAGCCTCAGCGTCAGTTACAGACCAGAGAGCCGCCTTCGCCACTGGTGTTCCTCCATATATCTACGCATTTCACCGCTACACATGGA

POSITIVE DOG: *Lactobacillus crispatus.*

AAAGTCACGGCTAACTACGTGCCAGCAGCCGCGGTAATACGTAGGTGGCAAGCGTTGTCCGGATTTATTGGGCGTAAAGCGAGCGCAGGCGGAAGAATAAGTCTGATGTGAAAGCCCTCGGCTTAACCGAGGAACTGCATCGGAAACTGTTTTTCTTGAGTGCAGAAGAGGAGAGTGGAACTCCATGTGTAGCGGTGGAATGCGTAGATATATGGAAGAACACCAGTGGCGAAGGCGGCTCTCTGGTCTGCAACTGACGCTGAGGCTCGAAAGCATGGGTAGCGAACAGGATTAGATACCCTGGTAGTCCATGCCGTAAACGATGAGTGCTAAGTGTTTGGGAGGTTTCCGCCTCTCAGTGCTGCAGCTAACGCATTAAGCACTCCGCCTGGGGAGTACGACCGCAAGGTTGAAACTCAAAGGAATTGACGGGGGCCCGCACAAGCGGTGGAGGCATGGTGGTTTAATTCGAAGCAACGCGAAGAACCCTTACCAGGTCTTGACATCTAGTGCCATTTGTAGAGATACAAAGTTCCCCTTCGGGGACGCTAAGACAGGTGGGTGCATGGCTGTCGTCAGCTCGTGTCCTGAGA

POSITIVE DOG: *Enterococcus faecium*

CCGACTTCCCAACTCGTTGTACTTCCCATTGGAACACGTGTGTAACCCAGGGCATAAGGGGGATGATGATTTGACGTCCTCCCCACCTTCCTCCGGTTTGTCACCGGCAGTCTTGGTAAAATGGCCAACTGAATGATGGCAACTAACAATAAGGGTTGCGCTCGTTGCGGGACTTAACCCAACATCTCACGACACGAACTGACGACCACCATGCACCACCTGGCACTTTGCCCCCGAAGGGGAAGCTCTATCTCTAGAGTGGTCAAAGGATGTCAAGACCTGGTAAGGGTCTTCCCGTTGCTTCCAATTAAACCACATGCTCCACCGCTTGGGCGGGCCCCCGTCAATTCCTTTGAATTTCAACCTTGCGGTCGTACTCCCCAGGGGGAATGGTTAATGGGTTAACTGCAGCACTGAAGGGCGGAAACCCTCCAACACTTAACACTCATCGTTTACGGCGTGGACTACCAAGGTATCTAATCCTGTTTGCTCCCCACGCTTTCGAGCCTCAGCGTCAGTTACAGACCAGAGAGCCGCCTTCGCCACTGGTGTTCCTCCATATATCTACGCATTTCACCGCTACAC

POSITIVE DOG: *Enterococcus gilvus.*

GATCGGCCACATTGGGACTGAGACACGACCCAAACTCTTACGGGAGGCAGCAGTAGGGAATCTTCGGCAATGGACGAAAGTCTGACCGAGCAACGCCGCGTGAGTGAAGAAGGTTTTCGGATCGTAAAACTCTGTTGTTAGAGAAGAACAAGGATGAGAGTAGAATGTTCATCCCTTGACGGTATCTAACCAGAAAGCCACGGCTAACTACGTGCCAGCAGCCGCGGTAATACGTAGGTGGCAAGCGTTGTCCGGATTTATTGGGCGTAAAGCGAGCGCAGGCGGTTTCTTAAGTCTGATGTGAAAGCCCCCGGCTCAACCGGGGAGGGTCATTGGAAACTGGGAAACTTGAGTGCAGAAGAGGAGAGTGGAATTCCATGTGTAGCGGTGAAATGCGTAGATATATGGAGGAACACCAGTGGCGAAGGCGGCTCTCTGGTCTGTAACTGACGCTGAGGCTCGAAAGCGTGGGGAGCAAACAGGATTAGATACCCTGGTAGTCCACGCCGTAAACGATGAGTGCTAAGTGTTGGAGGGTTTCCGCCCTTCAGTGCTGCAGCTAACGCATTAAGCACTCCGCCTGGGGAGTACGACCGCAAGGTTGAAACTCAAAGGAATTGACGGGGGCCCGCACAAGCGGTGGAAGCATGTGGTTTAATTCGAAGCAACGCGAAGAACCCTTACCAGGTCTTGACATCCTTTGACCACTCTAGAGAT

POSITIVE DOG: *Enterococcus gilvus*

GATCGTAAAACTCTGTTGTTAGAGAAGAACAAGGATGAGAGTAGAATGTTCATCCCTTGACcGGTATCTAAaCCAGAAAaGCCACGGCcTAACTACGTGCCAGCAGCCGCGGTAATACGTAGGTGGCAAGCGTTGTCCGGATTTATTGGGCGTAAAGCGAGCGCAGGCGGTTTCTTAAGTCTGATGTGAAAGCCCCCcgGGCTCAACCGGGGAGGGTCATTGGAAACTGGGAAACTTGAGTGCAGAAGAGGAGAGTGGAATTCCATGTGTAGCGGTGAAATGCGTAGATATATGGAGGAACACCAGTGGCGAAGGCGGCTCTCTGGTCTGTAACTGACGCTGAGGCTCGAAAGCGTGGGGAGCAAACAGGATTAGATACCCTGGTAGTCCACGCCGTAAACGATGAGTGCTAAGTGTTGGAGGGTTTCCGCCCTTCAGTGCTGCAGCTAACGCATTAAGCACTCCGCCTGGGGGAGTACGACCGCAAAGGTTGAAACTCAAAGGAATTGACGGGGGCCCGCACAAGCGGTGGGAGCATGTGGTTTAATTCGAAGCAACGCGAAGAACC

POSITIVE DOG: *Lactobacillus ingluviei.*

TAGCTTGTTGGTGGGGTAACGGCCTACCAAGGCGATGATGCGTAGCCGAGTTGAGAGACTGATCGGCCACAATGGGACTGAGACACGGCCCATACTCCTACGGGAGGCAGCAGTAGGGAATCTTCCACAATGGGCGCAAGCCTGATGGAGCAACACCGCGTGAGTGAAGAAGGGTTTCGGCTCGTAAAGCTCTGTTGTTGAAGAAGAATGTGCAAGAGAGTAACTGTTCTTGTATTGACGGTATTCAACCAGAAAGTCACGGCTAACTACGTGCCAGCAGCCGCGGTAATACGTAGGTGGCAAGCGTTATCCGGATTTATTGGGCGTAAAGCGAGCGCAGGCGGTTTTCTAAGTCTGATGTGAAAGCCTTCGGCTTAACCGAAGAAGTGCATCGGAAACTGGATGACTTGAGTGCAGAAGAGGGCAGTGGAACTCCATGTGTAGCGGTGGAATGCGTAGATATATGGAAGAACACCAGTGGCGAAGGCGGCTGCCTGGTCTGTAACTGACGCTGAGGCTCGAAAGCATGGGTAGCGAACAGGATTAGATACCCTGGTAGTCCATGCCGTAAACGATGAGTGCTAGGTGGTTTGGAGGGTTTCCGCCCTTCAGTGCCGGAGCTAACGCATTAAGCACTCCGCCTGGGGAGTACGACCGCAAGGTTGAAACTCAAGGAATTGACGGGGGCCCGCACAAGCGGTGGAAGCATTGGTGGGTTTAATTCGAAGCTACGCGAAGAACCCTTACCACGTCTTTGACATCTTTGCGCCAACCCTAGAAGATAGGGCGTTTCCCTTTCGG

POSITIVE DOG: *Enterococcus faecalis.*

ACCGCATAACAGTTTATGCCGCATGGCATAAGAGTGAAAGGCGCTTTCGGGTGTCGCTGATGGATGGACCCGCGGTGCATTAGCTAGTTGGTGAGGTAACGGCTCACCAAGGCCACGATGCATAGCCGACCTGAGAGGGTGATCGGCCACACTGGGACTGAGACACGGCCCAGACTCCTACGGGAGGCAGCAGTAGGGAATCTTCGGCAATGGACGAAAGTCTGACCGAGCAACGCCGCGTGAGTGAAGAAGGTTTTCGGATCGTAAAACTCTGTTGTTAGAGAAGAACAAGGACGTTAGTAACTGAACGTCCCCTGACGGTATCTAACCAGAAAGCCACGGCTAACTACGTGCCAGCAGCCGCGGTAATACGTAGGTGGCAAGCGTTGTCCGGATTTATTGGGCGTAAAGCGAGCGCAGGCGGTTTCTTAAGTCTGATGTGAAAGCCCCCGGCTCAACCGGGGAGGGTCATTGGAAACTGGGAGACTTGAGTGCAGAAGAGGAGAGTGGAATTCCATGTGTAGCGGTGAAATGCGTAGATATATGGAGGAACACCAGTGGCGAAGGCGGCTCTCTGGTCTGTAACTGACGCTGAGGCTCGAAAGCGTGGGGAGCAAACAGGATTAGATACCCTGGTAGTCCACGCCGTAAA

POSITIVE DOG: *Lactobacillus johnsonii.*

TTCGGCTCGTAAAGCTCTGTTGGgTAGTGAAGAAAGATAGAGGTAGTAACTGGCCTTTATTTGACGGgTAATTtACTTAGAAAGTCACGGCTAACTACGTGCCAGCAGCCGCGGTAATACGTAGGTGGCAAGCGTTGTCCGGATTTATTGGGCGTAAAGCGAGTGCAGGCGGTTCAATAAGTCTGATGTGAAAGCCTTCGGCTCAACCGGAGAATTGCATCAGAAACTGTTGAACTTGAGTGCAGAAGAGGAGAGTGGAACTCCATGTGTAGCGGTGGAATGCGTAGATATATGGAAGAACACCAGTGGCGAAGGCGGCTCTCTGGTCTGCAACTGACGCTGAGGCTCGAAAGCATGGGTAGCGAACAGGATTAGATACCCTGGTAGTCCATGCCGTAAACGATGAGTGCTAAGTGGTTGGGGAGGTTTCCGCCTCTCAGTGCTGCAGCTAACGCATTAAGCACTCCGCCTGGGGAGTACGACCGCAAGGTTGAAACTCAAaGGAATTGACGGGGGCCCGCACAAGCGGTGG

POSITIVE DOG: *Enterococcus faecalis.*

GTGAAGAAGGTTTTCGGATCGTAAAACTCTGTTGTTAGAGAAGAACAAGGgACGTTAGTAAaCTGAACGTCCCCTGACGGTATCTAACCcAGAAAGCCACGGCTAACTACGTGCCAGCAGCCGCGGTAATACGTAGGTGGCAAGCGTTGTCCGGATTTATTGGGCGTAAAGCGAGCGCAGGCGGTTTCTTAAGTCTGATGTGAAAGCCCCCGGCTCAACCGGGGAGGGTCATTGGAAACTGGGAGACTTGAGTGCAGAAGAGGAGAGTGGAATTCCATGTGTAGCGGTGAAATGCGTAGATATATGGAGGAACACCAGTGGCGAAGGCGGCTCTCTGGTCTGTAACTGACGCTGAGGCTCGAAAGCGTGGGGAGCAAACAGGATTAGATACCCTGGTAGTCCACGCCGTAAACGATGAGTGCTAAGTGTTGGAGGGTTTCCGCCCTTCAGTGCTGCAGCAAACGCATTAAGCACTCCGCCTGGGGAGTACGACCGCAAGGTTGAAACTCAAAGGAATTGACGGGGGCCCGCACAAGCGGTGGAGCAT
